# Human articular chondrocytes express 15-lipoxygenase-1 and -2: potential role in osteoarthritis

**DOI:** 10.1186/ar2652

**Published:** 2009-03-18

**Authors:** Nadir Chabane, Nadia Zayed, Mohamed Benderdour, Johanne Martel-Pelletier, Jean-Pierre Pelletier, Nicolas Duval, Hassan Fahmi

**Affiliations:** 1 Osteoarthritis Research Unit, Research Centre of the University of Montreal Hospital Center (CR-CHUM), Notre-Dame Hospital, Sherbrooke Street East, Montreal, Quebec H2L 4M1, Canada; 2 Department of Medicine, University of Montreal, Montreal, Quebec H2L 4M1, Canada; 3 Research Centre, Sacré-Coeur Hospital, Gouin Boulevard West, Montreal, Quebec H4J 1C5 Canada; 4 Centre de Convalescence, de Charmilles Pavillion, des Laurentides Boulevard, Montreal, Quebec H7M 2Y3 Canada

## Abstract

**Introduction:**

15-Lipoxygenases and their metabolites have been shown to exhibit anti-inflammatory and immunomodulatory properties, but little is known regarding their expression and function in chondrocytes. The objective of this study was to evaluate the expression of 15-lipoxygenase-1 and -2 in human articular chondrocytes, and to investigate the effects of their metabolites 13(S)-hydroxy octadecadienoic and 15(S)-hydroxyeicosatetraenoic acids on IL-1β-induced matrix metalloproteinase (MMP)-1 and MMP-13 expression.

**Methods:**

The expression levels of 15-lipoxygenase-1 and -2 were analyzed by reverse transcription PCR and Western blotting in chondrocytes, and by immunohistochemistry in cartilage. Chondrocytes or cartilage explants were stimulated with IL-1β in the absence or presence of 13(S)-hydroxy octadecadienoic and 15(S)-hydroxyeicosatetraenoic acids, and the levels of MMP-1 and MMP-13 protein production and type II collagen cleavage were evaluated using immunoassays. The role of peroxisome proliferator-activated receptor (PPAR)γ was evaluated using transient transfection experiments and the PPARγ antagonist GW9662.

**Results:**

Articular chondrocytes express 15-lipoxygenase-1 and -2 at the mRNA and protein levels. 13(S)-hydroxy octadecadienoic and 15(S)-hydroxyeicosatetraenoic acids dose dependently decreased IL-1β-induced MMP-1 and MMP-13 protein and mRNA expression as well as type II collagen cleavage. The effect on MMP-1 and MMP-13 expression does not require *de novo *protein synthesis. 13(S)-hydroxy octadecadienoic and 15(S)-hydroxyeicosatetraenoic acids activated endogenous PPARγ, and GW9662 prevented their suppressive effect on MMP-1 and MMP-13 production, suggesting the involvement of PPARγ in these effects.

**Conclusions:**

This study is the first to demonstrate the expression of 15-lipoxygenase-1 and -2 in articular chondrocytes. Their respective metabolites, namely 13(S)-hydroxy octadecadienoic and 15(S)-hydroxyeicosatetraenoic acids, suppressed IL-1β-induced MMP-1 and MMP-13 expression in a PPARγ-dependent pathway. These data suggest that 15-lipoxygenases may have chondroprotective properties by reducing MMP-1 and MMP-13 expression.

## Introduction

Osteoarthritis (OA) is the most common form of arthritis, accounting for a large proportion of disability in adults. The destruction of articular cartilage is a typical pathological characteristic of the disease [1,2]. and is believed to be largely mediated by proteases belonging to the matrix metalloproteinase (MMP) family of enzymes [3]. The MMPs can be classified into at least five main groups, including the collagenases (MMP-1, -8, and -13), the gelatinases (MMP-2 and -9), the stromelysins (MMP-3, -10, and -11), the matrilysins (MMP-7 and -26), and the membrane-bound-type MMPs (MMP-14, -15, -16, -17, -24, and -25). Among the MMPs, two collagenases, namely MMP-1 and MMP-13, are considered key players in the pathogenesis of OA because they have the unique ability to cleave most components of cartilage matrix, including collagen and aggrecan [3-5]. The expression levels of MMP-1 and MMP-13 are upregulated in arthritic tissues [6,7], and the pro-inflammatory cytokines IL-1β, tumor necrosis factor (TNF)-α, and IL-17, which are also upregulated in OA tissues, are known to induce strongly the production of both MMPs in articular chondrocytes [6-8]. Inhibition of MMP has been considered a therapeutic strategy in arthritis, but most clinical trials have yielded disappointing results [9-11]. Thus, identification of factors and pathways that modulate MMP-1 and MMP-13 expression in chondrocytes is critical to our understanding the pathogenesis of OA and may lead to the development of new therapeutic targets for the treatment of the disease.

Lipoxygenases (LOXs) are a family of enzymes that incorporate molecular oxygen at specific positions into unsaturated fatty acids. In human tissues, three major LOXs have been characterized and named according to the carbon position of arachidonic acid oxygenation [12,13]: 5-LOX, 12-LOX, and 15-LOX. Two different human 15-LOXs have been identified that differ in tissue distribution and substrate preferences. 15-LOX-1 is expressed in reticulocytes, eosinophils, skin, and macrophages [14,15]. 15-LOX-2 has been detected in prostate, lung, skin, and cornea [16]. 15-LOX-1 preferentially converts linoeic acid to 13(S)-hydroxy octadecadienoic acid (HODE), whereas 15-LOX-2 essentially converts arachidonic acid to 15(S)-hydroxyeicosatetraenoic acid (HETE) [16].

Several studies have documented that 15-LOXs and their metabolites exhibit anti-inflammatory and immunomodulatory properties. For instance, 15-HETE and 13-HODE were shown to inhibit the production of leukotriene-B_4 _and reactive oxygen species by stimulated neutrophils [17], and the production of IL-8 by colonic cells [18]. In addition, 15-LOX metabolites suppress the production of TNF-α, a key cytokine in the pathogenesis of arthritis [19,20], and mediate the effects of the T-helper-2 cytokine IL-4 [21,22]. The 15-LOX metabolites 15-HETE and 13-HODE are also ligands for the peroxisome proliferator-activated receptor (PPAR)γ [23,24]. PPARγ is a unique member of the ligand-dependent nuclear receptor family that has been implicated in the modulation of critical aspects of development and homeostasis. We and others have shown that PPARγ activation inhibits the expression of a number of genes involved in the pathogenesis of OA, including IL-1β, TNF-α, MMP-1, MMP-13, inducible nitric oxide synthase, and microsomal prostaglandin E synthase-1 [25-28], and is protective in animal models of OA [29].

The expression of 15-LOXs and the roles played by their metabolites have been characterized in various tissues and cell types [12-16]. However, little is known regarding the expression and function of 15-LOXs in human cartilage. This study was undertaken to investigate the expression of 15-LOXs in human articular OA chondrocytes and to define the effect of their metabolites 15-HETE and 13-HODE on IL-1β-induced MMP-1 and MMP-13 production. We provide evidence that both 15-LOX-1 and 15-LOX-2 are expressed in human OA chondrocytes. We also demonstrate that 13-HODE and 15-HETE suppressed IL-1β-induced MMP-1 and MMP-13 expression and type II collagen cleavage. These data suggest that 15-LOXs may play a role in preventing the cartilage destruction observed in OA.

## Materials and methods

### Reagents

Recombinant human IL-1β was obtained from Genzyme (Cambridge, MA, USA), and recombinant human TNF-α and recombinant human IL-17 from R&D Systems (Minneapolis, MN, USA). GW9662, 13(S)-HODE, 15(S)-HETE, anti-15-LOX-1 and 15-LOX-2 antibodies were from Cayman Chemical Co. (Ann Arbor, MI, USA). Cycloheximide was from Sigma-Aldrich Canada (Oakville, Ontario, Canada), and Dulbecco's modified Eagle's medium (DMEM), penicillin and streptomycin, fetal calf serum (FCS), and TRIzol^® ^reagent were from Invitrogen (Burlington, Ontario, Canada). All other chemicals were purchased from either Sigma-Aldrich Canada or Bio-Rad (Mississauga, Ontario, Canada).

### Specimen selection and chondrocyte culture

Human OA cartilage samples from femoral condyles and tibial plateaus were obtained from OA patients undergoing total knee replacement (n = 23; mean ± standard deviation [SD] age 68 ± 13 years). All OA patients were diagnosed in accordance with the criteria developed by the American College of Rheumatology Diagnostic Subcommittee for OA [30]. At the time of surgery, the patients had symptomatic disease requiring medical treatment in the form of nonsteroidal anti-inflammatory drugs or selective cyclo-oxygenase-2 inhibitors. Patients who had received intra-articular injections of steroids were excluded. The Clinical Research Ethics Committee of the Notre-Dame Hospital approved the study protocol and the use of human articular tissues.

Chondrocytes were released from cartilage by sequential enzymatic digestion, as previously described [26]. In brief, this consisted of 2 mg/ml pronase for 1 hour followed by 1 mg/ml collagenase (type IV; Sigma-Aldrich) for 6 hours at 37°C in DMEM and antibiotics (100 U/ml penicillin and 100 μg/ml streptomycin). The digested tissue was briefly centrifuged and the pellet was washed. The isolated chondrocytes were seeded at high density in tissue culture flasks and cultured in DMEM supplemented with 10% heat-inactivated FCS.

Confluent chondrocytes were detached by trypsinization, seeded at 3.5 × 10^5 ^cells per well in 12-well culture plates (Costar, Corning, NY, USA) or at 7 × 10^5 ^cells per well in six-well culture plates in DMEM supplemented with 10% FCS, and cultivated at 37°C for 48 hours. Cells were washed and incubated for an additional 24 hours in DMEM containing 0.5% FCS, before stimulation with either IL-1β alone or in combination with 13-HODE or 15-HETE. 13-HODE and 15-HETE, supplied in ethanol at 1 mg/ml, were air-dried and dissolved in dimethyl sulfoxide at 10 mg/ml. Control cells were treated with the highest concentration of dimethyl sulfoxide (0.14%) as vehicle control. In another set of experiments, chondrocytes were pretreated for 30 minutes with vehicle, cycloheximide, or GW9662 before stimulation. The levels of MMP proteins released in supernatants were determined 24 hours after stimulation, whereas MMP mRNA levels were determined at 8 hours. Only first passaged chondrocytes were used.

### RNA extraction and PCR analyses

Total RNA was isolated using the TRIzol^® ^reagent (Invitrogen), in accordance with the manufacturer's instructions. To remove contaminating DNA, isolated RNA was treated with RNase-free DNase I (Ambion, Austin, TX, USA). The RNA was quantitated using the RiboGreen RNA quantitation kit (Molecular Probes, Eugene, OR, USA), dissolved in diethylpyrocarbonate-treated water and stored at -80°C until use. One microgram of total RNA was reverse transcribed using Moloney murine leukemia virus reverse transcriptase (Fermentas, Burlington, Ontario, Canada), as detailed in the manufacturer's guidelines. One-fifth of the reverse transcriptase reaction was analyzed by traditional PCR or real-time quantitative PCR. The following primers were used: 15-LOX-1, sense 5'-TTGGTTATTTCAGCCCCCATC-3' and antisense 5'-TGTGTTCACTGGGTGCAGAGA-3'; 15-LOX-2, sense 5'-GCATCCACTGATTGGACCTT-3' and antisense 5'-GCTGGCCTTGAACTTCTGAC-3'; MMP-1, sense 5'-CTGAAAGTGACTGGGAAACC-3' and antisense 5'-AGAGTTGTCCCGATGATCTC-3'; MMP-13, sense 5'-CTT AGA GGT GAC TGG CAA AC-3' and antisense 5'-GCC CAT CAA ATG GGT AGA AG-3'; and glyceraldehyde-3-phosphate dehydrogenase (GAPDH), sense 5'-CAGAACATCATCCCTGCCTCT-3' and antisense 5'-GCTTGACAAAGTGGTCGTTGAG-3'.

Quantitative PCR analysis was performed in a total volume of 50 μl containing template DNA, 200 nmol/l of sense and antisense primers, 25 μl of SYBR^® ^Green master mix (QIAGEN, Mississauga, Ontario, Canada), and uracil-N-glycosylase (UNG; 0.5 units; Epicentre Technologies, Madison, WI, USA). After incubation at 50°C for 2 minutes (UNG reaction) and at 95°C for 10 minutes (UNG inactivation and activation of the AmpliTaq Gold enzyme), the mixtures were subjected to 40 amplification cycles (15 seconds at 95°C for denaturation and 1 minute for annealing and extension at 60°C). Incorporation of SYBR^® ^Green dye into PCR products was monitored in real time using a GeneAmp 5700 Sequence detection system (Applied Biosystems, Foster City, CA, USA), allowing determination of the threshold cycle (C_T_) at which exponential amplification of PCR products begins. After PCR, dissociation curves were generated with one peak indicating the specificity of the amplification. A threshold cycle (C_T _value) was obtained from each amplification curve using the software provided by the manufacturer (Applied Biosystems).

Relative mRNA expression in chondrocytes was determined using the ΔΔC_T _method, as detailed in the manufacturer's guidelines (Applied Biosystems). A ΔC_T _value was first calculated by subtracting the C_T _value for the housekeeping gene GAPDH from the C_T _value for each sample. A ΔΔC_T _value was then calculated by subtracting the ΔC_T _value of the control (unstimulated cells) from the ΔC_T _value of each treatment. Fold changes compared with the control were then determined by raising 2 to the power of -ΔΔC_T_. Each PCR reaction generated only the expected specific amplicon, as shown by the melting temperature profiles of the final product and by gel electrophoresis of test PCR reactions. Each PCR was performed in triplicate on two separate occasions for each independent experiment. In conventional PCR, the mixtures were incubated at 95°C for 1 minute followed by 35 cycles each at 94°C/30 seconds and 60°C/1 minute, with a final elongation step at 60°C/8 minutes. Controls for reverse transcription and PCR amplifications were included. PCR product (10 μl/50 μl) reactions were separated on a 1.8% agarose gel and stained with ethidium bromide.

### Western blot analysis

Chondrocytes were lysed in ice-cold lysis buffer (50 mmol/l Tris-HCl [pH 7.4], 150 mmol/l NaCl, 2 mmol/l EDTA, 1 mmol/l PMSF, 10 μg/ml each of aprotinin, leupeptin, and pepstatin, 1% NP-40, 1 mmol/l Na_3_VO_4_, and 1 mmol/l NaF). Lysates were sonicated on ice and centrifuged at 12,000 rpm for 15 minutes. The protein concentration of the supernatant was determined using the bicinchoninic acid method (Pierce, Rockford, IL, USA). Twenty micrograms of total cell lysate was subjected to SDS-PAGE and electrotransferred to a nitrocellulose membrane (Bio-Rad). After blocking in 20 mmol/l Tris-HCl (pH 7.5) containing 150 mmol/l NaCl, 0.1% Tween 20, and 5% (weight/volume) nonfat dry milk, blots were incubated overnight at 4°C with the primary antibody and washed with a Tris buffer (Tris-buffered saline [pH 7.5], with 0.1% Tween 20). The blots were then incubated with horseradish peroxidase-conjugated secondary antibody (Pierce), washed again, incubated with SuperSignal Ultra Chemiluminescent reagent (Pierce), and exposed to Kodak X-Omat film (Eastman Kodak Ltd, Rochester, NY, USA).

### Immunohistochemistry

Cartilage specimens were processed for immunohistochemistry, as described previously [26]. The specimens were fixed in 4% paraformaldehyde and embedded in paraffin. Sections (5 μm) of paraffin-embedded specimens were deparaffinized in toluene, and dehydrated in a graded series of ethanol. The specimens were then pre-incubated with chondroitinase ABC (0.25 U/ml in phosphate-buffered saline [PBS; pH 8.0]) for 60 minutes at 37°C, followed by a 30-minute incubation with Triton X-100 (0.3%) at room temperature. Slides were then washed in PBS followed by 2% hydrogen peroxide/methanol for 15 minutes. They were further incubated for 60 minutes with 2% normal serum (Vector Laboratories, Burlingame, CA, USA) and overlaid with primary antibody for 18 hours at 4°C in a humidified chamber. Each slide was washed three times in PBS (pH 7.4) and stained using the avidin-biotin complex method (Vectastain ABC kit; Vector Laboratories). The color was developed with 3,3'-diaminobenzidine (Vector Laboratories) containing hydrogen peroxide. The slides were counterstained with eosin. The specificity of staining was evaluated by substituting the primary antibody with nonimmune IgG (Chemicon, Temecula, CA, USA) at the same concentration as the primary antibody. The evaluation of positive-staining chondrocytes was performed using our previously published method [26]. For each specimen, six microscopic fields were examined under 40× magnification. The total number of chondrocytes and the number of chondrocytes staining positive were evaluated, and the results were expressed as the percentage of chondrocytes staining positive (cell score).

### Plasmids and transient transfection

The PPRE-luciferase construct containing three PPAR-responsive elements (PPREs) cloned upstream of the thymidine kinase promoter (PPRE-Tk-luciferase) was generously provided by Dr CK Glass (University of California, San Diego, CA, USA). β-Galactosidase reporter vector under the control of SV40 promoter (pSV40-β-galactosidase) was from Promega (Madison, WI, USA). Transient transfection experiments were performed using FuGene-6 (1 μg DNA: 4 μl FuGene 6; Roche Applied Science, Laval, Quebec, Canada), in accordance with the manufacturer's recommended protocol. Briefly, chondrocytes were seeded 24 hours before transfection at a density of 6 × 10^5 ^cells/well in six-well plates and transiently transfected with 1 μg of the reporter construct and 0.5 μg of the internal control pSV40-β-galactosidase. Six hours later, the cells were rinsed in PBS and changed to medium containing 0.5% FCS for an additional 18 hours. The cells were then treated with increasing concentrations of 13-HODE or 15-HETE for 18 hours. In these conditions, transfection efficiency typically ranges between 40% and 50%. After harvesting, luciferase activity was determined and normalized to β-galactosidase activity. All of the transfection experiments were repeated at least three times in duplicate.

### Matrix metalloproteinase-1 and -13 determination

The levels of MMP-1 and MMP-13 in conditioned media were determined by specific ELISAs (R&D Systems Inc, Minneapolis, MN, USA). All measurements were performed in duplicate.

### Extraction and assay for cleavage of type II collagen

Cartilage explants were digested to extract cleaved type II collagen, as previously described [31]. Briefly, after treatment the harvested cartilage was incubated overnight at 37°C with 1.0 mg/50 mg cartilage of α-chymotrypsin in 50 mmol/l Tris-HCl (pH 7.6; with the following proteinase inhibitors: 1 mmol/l EDTA, 1 mmol/l iodoacetamide, and 10 μg/ml pepstatin A). After the α-chymotrypsin activity was inhibited with N-tosyl-L-phenylalanine-chloromethyl ketone (Sigma) for 20 minutes, the samples were centrifuged and the supernatants assayed for type II collagen degradation using a C2C ELISA kit (IBEX, Montreal, Quebec, Canada).

### Statistical analysis

Data are expressed as the mean ± SD. Statistical significance was assessed using the two-tailed Student's *t*-test. *P *values less than 0.05 were considered statistically significant.

## Results

### Human OA articular chondrocytes express both 15-LOX-1 and -2

**Figure 1:**
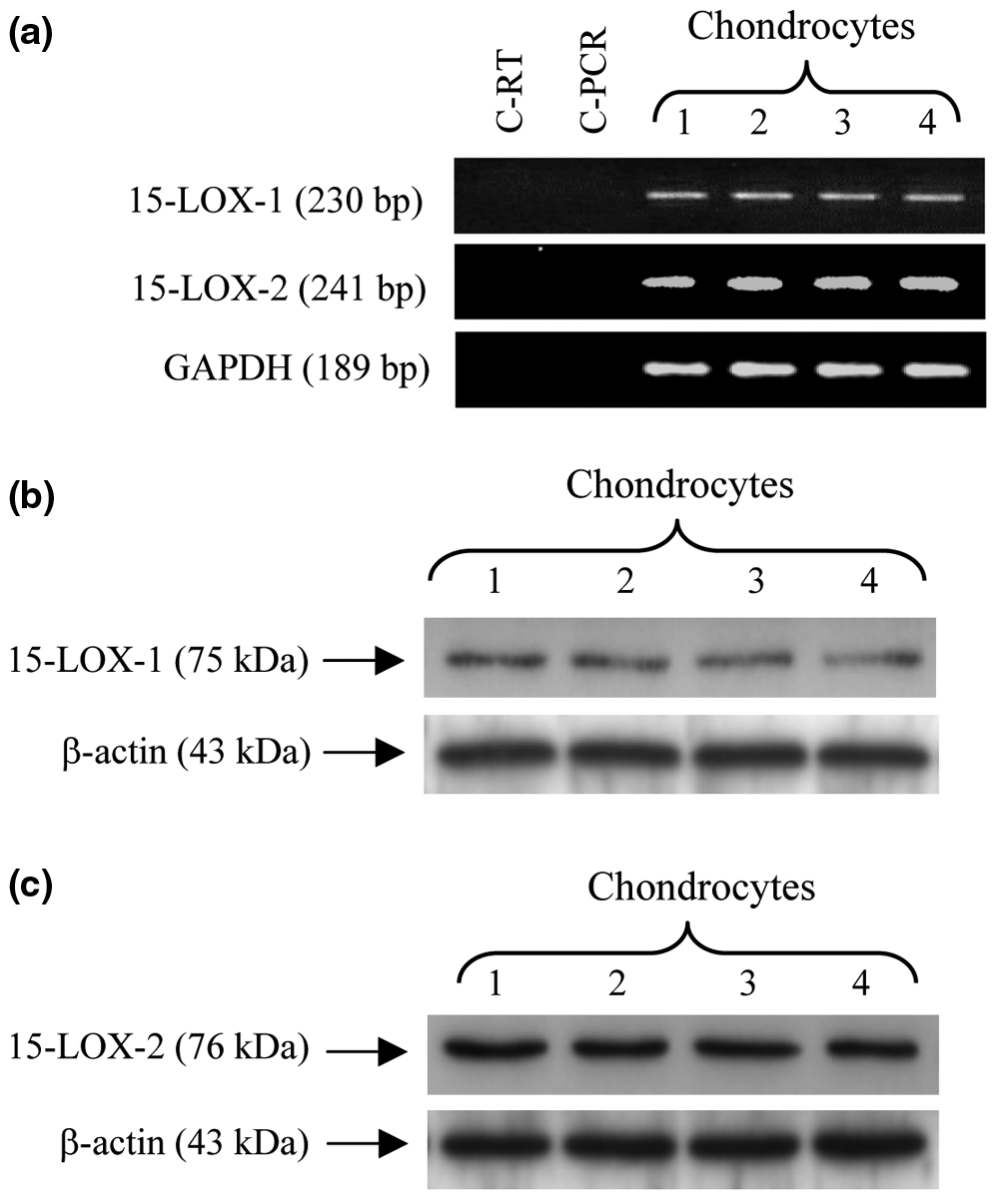
Human articular chondrocytes express both 15-LOX-1 and 15-LOX-2. **(a) **Chondrocytes were isolated from OA knee cartilage and maintained as monolayer culture for 7 to 10 days. Total RNA was prepared, reverse transcribed into cDNA, and processed for PCR using specific primers for 15-LOX-1, 15-LOX-2, and GAPDH. PCR products were resolved on a 1.8% agarose gel and stained with ethidium bromide. C-RT and C-PCR are negative controls for the reverse transcription and PCR reaction, respectively. **(b, c) **Chondrocytes were isolated from OA knee cartilage and lysates were prepared after 7 to 10 days in culture. Samples with equal amounts of total proteins (20 μg per lane) were immunoblotted with specific anti-15-LOX-1 (panel b) and anti-15-LOX-2 (panel c) antibodies (upper sections). The blots were stripped and reprobed with a specific anti-β-actin antibody (lower sections). bp, base pairs; GAPDH, glyceraldehyde-3-phosphate dehydrogenase; LOX, lipoxygenase; OA, osteoarthritis.

To investigate whether human articular chondrocytes express 15-LOX-1 and -2, total RNA from cultured chondrocytes, derived from four different OA patients, was subjected to reverse transcription PCR analysis using specific primers for 15-LOX-1 and -2. As shown in Figure 1a, the expression of 15-LOX-1 and -2 mRNAs was detected in the four chondrocyte preparations. No PCR products were obtained with control reactions performed in the absence of the cDNA or reverse transcriptase (Figure 1a). To further confirm the expression of 15-LOX-1 and -2 in chondrocytes, we analyzed their expression at the protein level. Western blot analysis with total protein extracts revealed the presence of both isoforms in all examined chondrocyte preparations (Figure 1b, c).

**Figure 2:**
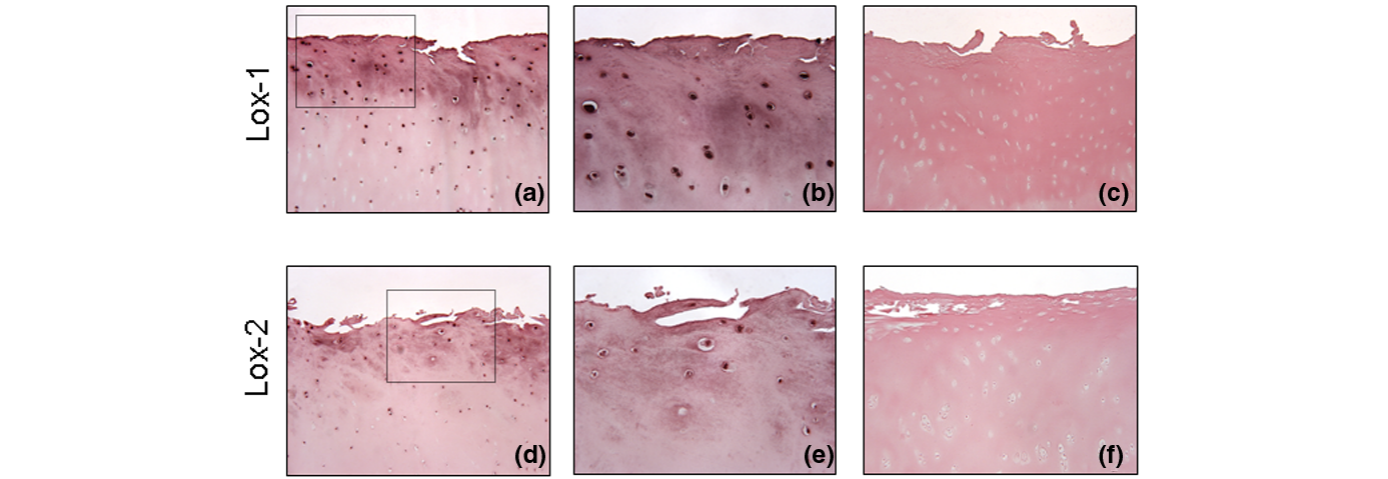
Expression of 15-LOX-1 and 15-LOX-2 in human OA cartilage. Representative immunostaining of human osteoarthritis (OA) cartilage for **(a) **15-LOX-1 and **(d) **15-LOX-2. **(b, e) **Higher magnification views of the area indicated within the broken line rectangle in panels a and d, respectively. **(c, f) **Cartilage treated with nonimmune control IgG at the same concentration as the primary antibody (control for staining specificity). (Magnification: ×100 for panels a, c, d and f; ×250 for panels b and e). The results are representative of four separate experiments performed with cartilage samples from four different donors. LOX, lipoxygenase.

To examine whether chondrocytes express 15-LOX-1 and -2 *in vivo*, we performed immunohistochemical analysis using OA cartilage. The positive immunostaining for 15-LOX-1 (Figure 2a) and 15-LOX-2 (Figure 2d) was located mainly in the superficial and intermediate zones of the cartilage. Statistical evaluation of the cell score revealed lower immunostaining for 15-LOX-1 (mean ± SD: 36.2% ± 17.6%) than for 15-LOX-2 (mean ± SD: 43.7% ± 19.2%), but these differences were not significant. The specificity of staining was confirmed using nonimmune control IgG (Figure 2c, f). These observations demonstrate the *in vivo *expression of 15-LOX-1 and -2 proteins in OA cartilage.

### 13-HODE and 15-HETE inhibited IL-1β-induced MMP-1 and MMP-13 expression in chondrocytes

**Figure 3:**
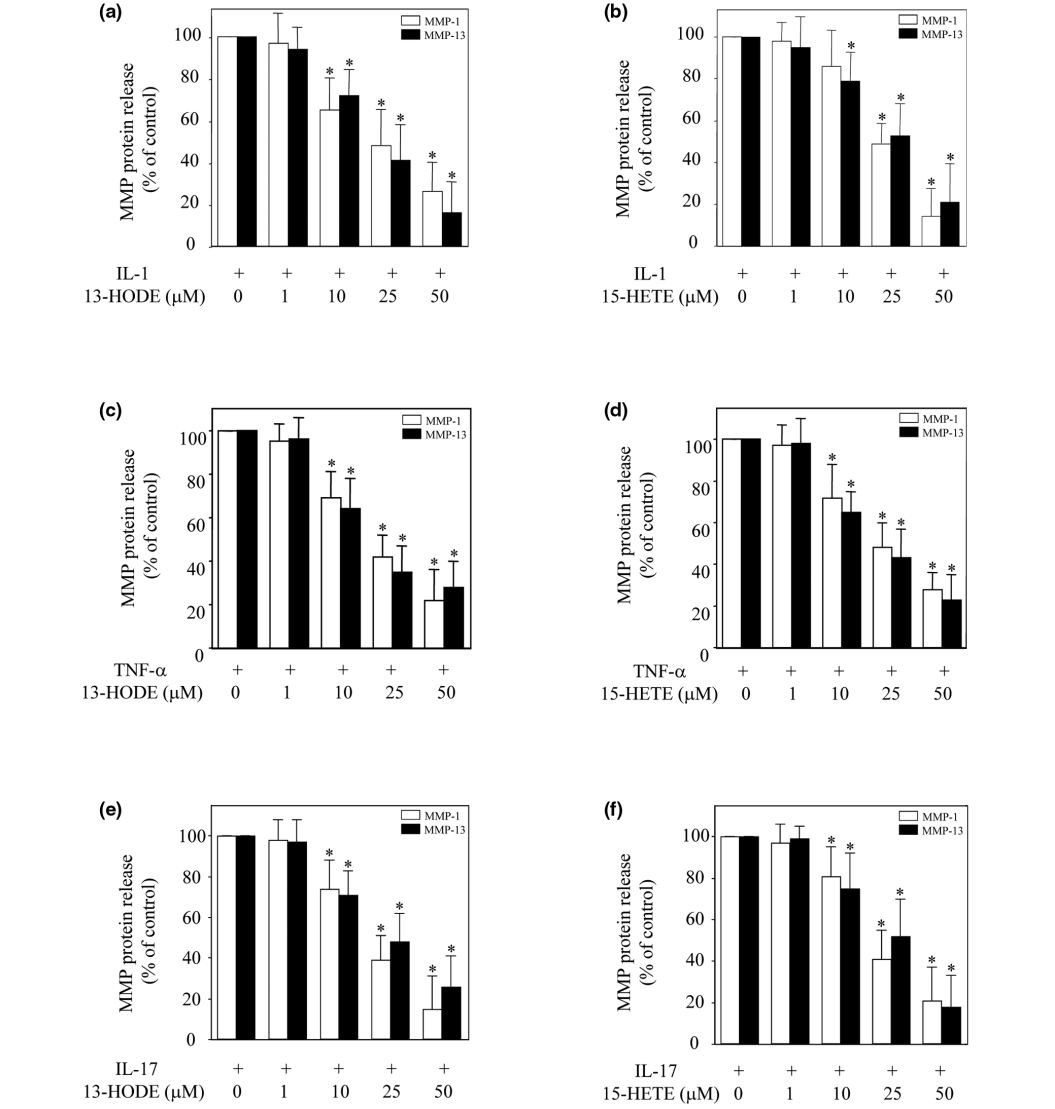
13-HODE and 15-HETE downregulate induction of MMP-1/MMP-13 protein synthesis by IL-1β, TNF-α and IL-17. **(a, b) **Chondrocytes were stimulated with IL-1β (100 pg/ml), **(c, d) **TNF-α (0.1 ng/ml), or **(e, f) **IL-17 (10 ng/ml) in the presence of vehicle (dimethyl sulfoxide at a maximum concentration of 0.14%) or increasing concentrations of 13-HODE (panels a, c, and e) or 15-HETE (panels b, d, and f) for 24 hours. The levels of MMP-1 and MMP-13 proteins in conditioned media were measured using ELISA. Results are expressed as the percentage of control, considering 100% as the value of cells treated with IL-1β, TNF-α or IL-17 alone, and are the mean ± standard deviation of at least three independent experiments. *P < 0.05 versus cells treated with IL-1β, TNF-α, or IL-17 alone. HETE, hydroxyeicosatetraenoic acid; HODE, hydroxy octadecadienoic acid; MMP, matrix metalloproteinase; TNF, tumor necrosis factor.

To examine the effects of 15-LOX-1 and -2 metabolites on MMP-1 and MMP-13 release, chondrocytes were stimulated with IL-1β in the absence or presence of increasing concentrations of 13-HODE or 15-HETE, and the levels of MMP-1 and MMP-13 proteins in conditioned media were determined by ELISA. As shown in Figure 3a, b, the production of MMP-1 and MMP-13 was dose dependently reduced in the presence of 13-HODE or 15-HETE. The concentrations of 13-HODE and 15-HETE utilized did not affect chondrocyte viability, as judged using the MTT (3- [4,5-dimethylthiazol-2-yl]-2,5-diphenyltetrazolium bromide) assay (data not shown). Taken together, these findings suggest that 15-LOX metabolites may constitute novel endogenous negative regulators of MMP-1 annd MMP-13 expression in chondrocytes.

In addition to IL-1, the pro-inflammatory cytokines TNF-α and IL-17 also contribute to the pathogenesis of OA and are potent inducers of MMP-1 and MMP-13. Therefore, we examined whether 13-HODE and 15-HETE could also attenuate TNF-α and IL-17-induced MMP-1 and MMP-13 production in chondrocytes. As shown in Figure 3c–e, the induction of MMP-1 and MMP-13 production by TNF-α or IL-17 was dose dependently diminished in the presence of 13-HODE or 15-HETE. These data suggest that the suppressive effect of 13-HODE and 15-HETE is not specific to IL-1, and is independent of the nature of the stimulus that triggers MMP-1 and MMP-13 production.

### 13-HODE and 15-HETE suppress IL-1-induced type II collagen cleavage

**Figure 4:**
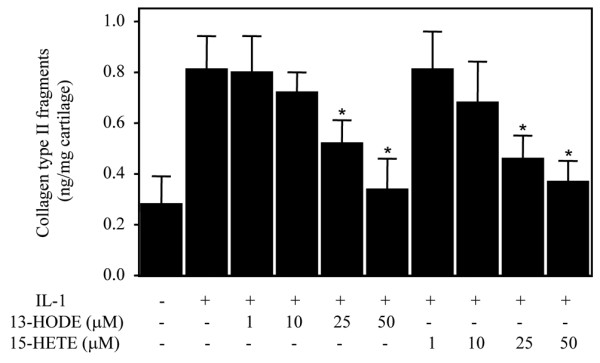
13-HODE and 15-HETE downregulate IL-1β-induced type II collagen degradation cleavage. Cartilage explants were stimulated with 1 ng/ml IL-1β in the presence of the control vehicle dimethyl sulfoxide or increasing concentrations of 13-HODE or 15-HETE for 5 days. Type II collagen degradation was assessed by quantification of C2C epitopes of type II collagen in cartilage explants. Data are the mean ± standard deviation of three independent experiments. *P < 0.05 versus cartilage explants treated with IL-1β alone. HETE, hydroxyeicosatetraenoic acid; HODE, hydroxy octadecadienoic acid.

Next, we assessed the effects of 13-HODE and 15-HETE on IL-1-induced type II collagen cleavage. Cartilage explants were treated with IL-1β in the absence or presence of increasing concentrations of 13-HODE or 15-HETE for 5 days, and type II collagen degradation was determined using a specific commercial kit that measures C2C epitopes of type II collagen. As shown in Figure 4, treatment with 13-HODE or 15-HETE dose-dependently prevented IL-1-induced type II collagen cleavage.

### Suppression of IL-1β-induced MMP-1 and MMP-13 expression by 13-HODE and 15-HETE does not require *de novo *protein synthesis

**Figure 5:**
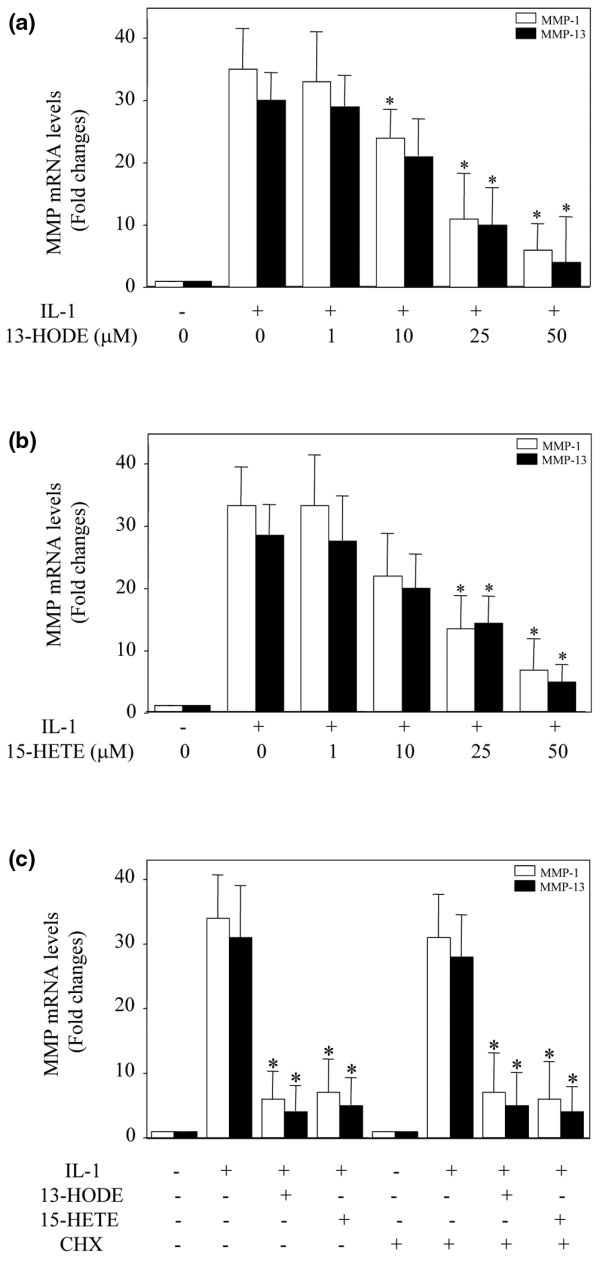
Downregulation of IL-1β-induced MMP-1/MMP-13 expression by 13-HODE and 15-HETE does not require *de novo *protein synthesis. **(a, b) **Chondrocytes were treated with 100 pg/ml IL-1β in the presence of the control vehicle dimethyl sulfoxide or increasing concentrations of 13-HODE (panel a) or 15-HETE (panel b) for 8 hours. **(c) **Chondrocytes were pretreated with control vehicle dimethyl sulfoxide or cycloheximide (10 μg/ml) for 30 minutes before stimulation with 100 pg/ml IL-1β in the absence or presence of 50 μmol/l 13-HODE or 15-HETE for 8 hours. Total RNA was isolated, reverse transcribed into cDNA, and MMP-1 and MMP-13 mRNAs were quantified using real-time PCR. The housekeeping gene GAPDH was used for normalization. All experiments were performed in triplicate, and negative controls without template RNA were included in each experiment. Results are expressed as fold changes, considering 1 as the value of untreated cells, and are the mean ± standard deviation of three independent experiments. *P < 0.05 versus cells treated with IL-1β alone. CHX, cycloheximide; GAPDH, glyceraldehyde-3-phosphate dehydrogenase; HETE, hydroxyeicosatetraenoic acid; HODE, hydroxy octadecadienoic acid; MMP, matrix metalloproteinase; TNF, tumor necrosis factor.

To investigate the effects of 13-HODE and 15-HETE on IL-1β-induced MMP-1 and MMP-13 mRNA expression, we used real-time PCR. Consistent with their effects on MMP-1 and MMP-13 protein production, 13-HODE and 15-HETE dose-dependently suppressed IL-1β-induced MMP-1 and MMP-13 mRNA expression (Figure 5a, b), suggesting that these effects occur at the transcriptional level.

To evaluate whether the effect of 13-HODE and 15-HETE on IL-1β-induced MMP-1 and MMP-13 expression is direct or indirect, we tested the impact of the protein synthesis inhibitor cycloheximide. Chondrocytes were pretreated with cycloheximide for 30 minutes and stimulated with IL-1β alone or in combination with either 13-HODE or 15-HETE for 8 hours. The levels of MMP-1 and MMP-13 mRNAs were analyzed by real-time PCR. As shown in Figure 5c, pretreatment with cycloheximide did not affect 13-HODE and 15-HETE-mediated inhibition of IL-1β-induced MMP-1 and MMP-13 expression, suggesting that their effect was a direct primary effect through pre-existing factors and was not dependent on *de novo *protein synthesis.

### 13-HODE and 15-HETE suppressed IL-1β-induced MMP-1 and MMP-13 production in a PPARγ dependent manner

**Figure 6:**
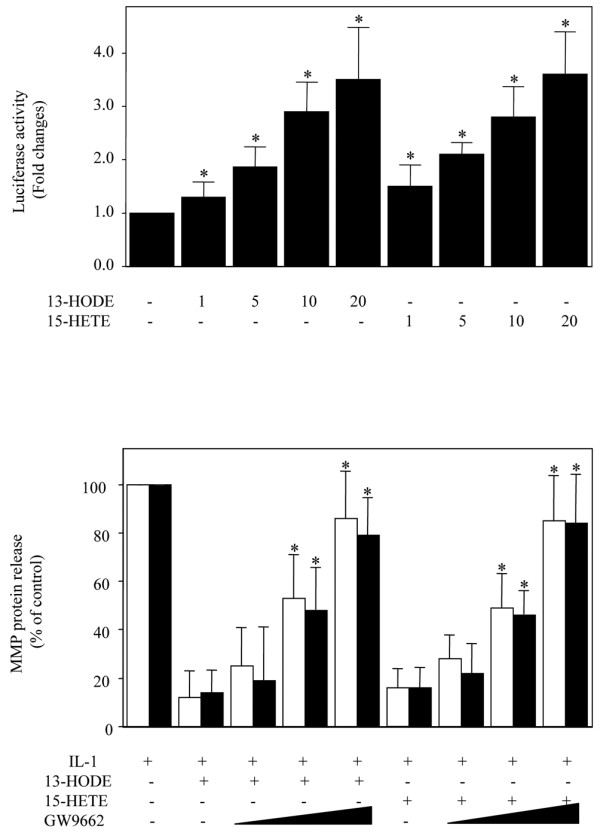
13-HODE and 15-HETE suppressed IL-1β-induced MMP-1/MMP-13 production in a PPARγ dependent manner. **(a) **13-HODE and 15-HETE activate endogenous PPARγ in human chondrocytes. Chondrocytes were transiently transfected with a reporter construct containing three copies of a consensus PPRE placed upstream from the Tk-luciferase reporter (PPRE_3_-Tk-Luc) along with the internal control pSV40-β-gal using FuGene 6 transfection reagent. Six hours later, the cells were washed and changed to medium containing 0.5% fetal calf serum for an additional 18 hours. Transfected cells were then treated with the control vehicle dimethyl sulfoxide or increasing concentrations of 13-HODE or 15-HETE for 18 hours. Luciferase activity values were determined and normalized to β-galactosidase activity. Results are expressed as fold changes, considering 1 as the value of unstimulated cells, and are the mean ± standard deviation of three independent experiments. *P < 0.05 versus unstimulated cells. **(b) **PPARγ antagonist (GW9662) prevented the suppressive effect of 13-HODE and 15-HETE on IL-1β-induced MMP-1 and MMP-13 release. Chondrocytes were pretreated with increasing concentrations (1, 5, and 10 μmol/l) of GW9662 for 30 minutes. Then, the cells were treated with or without IL-1β (100 pg/ml) for 24 hours in the absence or the presence of 50 μmol/l 13-HODE (panel a) or 50 μmol/l 15-HETE (panel b). The levels of MMP-1 and MMP-13 proteins in conditioned media were measured using ELISA. Results are expressed as the percentage of control, considering 100% as the value of cells treated with IL-1β alone, and are the mean ± standard deviation of four independent experiments. *P < 0.05 versus cells treated with IL-1β and 13-HODE or 15-HETE. HETE, hydroxyeicosatetraenoic acid; HODE, hydroxy octadecadienoic acid; MMP, matrix metalloproteinase; PPAR, peroxisome proliferator-activated receptor; PPRE, peroxisome proliferator-activated receptor-responsive element.

The 15-LOX metabolites 13-HODE and 15-HETE are ligands for PPARγ, and PPARγ activation was reported to suppress IL-1β-induced MMP-1 and MMP-13 production [26,27]. To test the possibility that PPARγ is involved in the suppressive effect of 13-HODE and 15-HETE on MMP-1 and MMP-13 production, we first examined their effects on the transcriptional activity of endogenous PPARγ in chondrocytes. Chondrocytes were transiently transfected with a luciferase reporter construct containing three copies of a consensus PPRE, and treated with increasing concentrations of 13-HODE and 15-HETE. As illustrated in Figure 6a, treatment with 13-HODE and 15-HETE dose dependently increased the activity of the synthetic promoter. These data confirm the presence of inducible PPARγ-dependent transcriptional responses in chondrocytes. Next, we examined the effect of GW9662, a selective and irreversible PPARγ antagonist. Chondrocytes were pre-incubated with increasing concentrations of GW9662 before addition of 13-HODE or 15-HETE and were subsequently stimulated with IL-1β. As shown in Figure 6b, GW9662 dose-dependently relieved the suppressive effect of 13-HODE and 15-HETE on IL-1β-induced MMP-1 and MMP-13 protein production. Taken together, these results strongly suggest that 13-HODE and 15-HETE inhibit IL-1β-induced MMP-1 and MMP-13 production through a PPARγ-dependent mechanism.

## Discussion

In the present study, we report for the first time that articular OA chondrocytes express 15-LOX-1 and -2. Treatment with 13-HODE and 15-HETE, the major products of 15-LOX-1 and -2, respectively, suppressed IL-1β-induced MMP-1 and MMP-13 expression and type II collagen degradation. Taken together, these findings strongly suggest a chondroprotective role for 15-LOXs by negatively regulating the expression of MMP-1 and MMP-13.

In addition to their chondroprotective properties observed in this study, 15-LOX metabolites were shown to exhibit potent anti-inflammatory effects. For instance, 15-HETE inhibits polymorphonuclear neutrophil degranulation and superoxide production elicited by N-formylmethionylleucylphenylalaline, platelet-activating factor and leukotriene B_4 _[17]. In addition, 15-HETE prevents polymorphonuclear neutrophil migration across IL-1β or TNF-α-activated endothelium [32] and TNF-α-induced expression of several adhesion molecules, including intercellular adhesion molecule-1, vascular cell adhesion molecule-1 and E-selectin [33]. On the other hand, 13-HODE attenuates the production of reactive oxygen species in macrophages [34], the production of IL-8 in colonic epithelial cells [18], and the ability of dendritic cells to activate interferon-γ secretion by T lymphocytes [35]. Moreover, 13-HODE and 15-HETE were shown to mediate the suppressive effect of the anti-inflammatory cytokine IL-4 on inducible nitric oxide synthase expression in macrophages [21] and IL-2 production in T lymphocytes [22]. In addition to 13-HODE and 15-HETE formation, 15-LOXs are involved in the generation of the potent anti-inflammatory molecules lipoxins, resolvings, and protectins [36]. Thus, 15-LOXs can dampen inflammation through production of distinct classes of anti-inflammatory and pro-resolution lipid mediators.

The protective effect of 15-LOXs is further supported by results from studies using transgenic animals. Over-expression of 15-LOX in rabbits reduced inflammation and tissue damage in atherosclerosis [37] and peritonitis [38]. In rats, over-expression of 15-LOX suppressed renal inflammation and preserved organ function in experimental glomerulonephritis [39]. These data, together with our findings that 15-LOX metabolites block MMP production, suggest that these lipids may have protective effects in OA *in vivo*. Further studies using cartilage-specific 15-LOX-null mice will be required to elucidate the role of 15-LOXs in cartilage integrity and the pathogenesis of OA.

Several factors are known to modulate 15-LOX expression. For instance, IL-4 and IL-13, increase the expression of 15-LOX-1 and -2 in a number of cell types, including monocytes/macrophages, T lymphocytes and several cancer cell lines [40-45]. Moreover, chromatin modifications that play pivotal roles in the regulation of gene expression were reported to modulate 15-LOX expression. Histone acetylation appears to upregulate 15-LOX expression [46] whereas DNA methylation downregulates 15-LOX expression [47]. Whether these factors and conditions contribute to the modulation of 15-LOX expression in chondrocytes is among our ongoing research projects.

**Figure 7:**
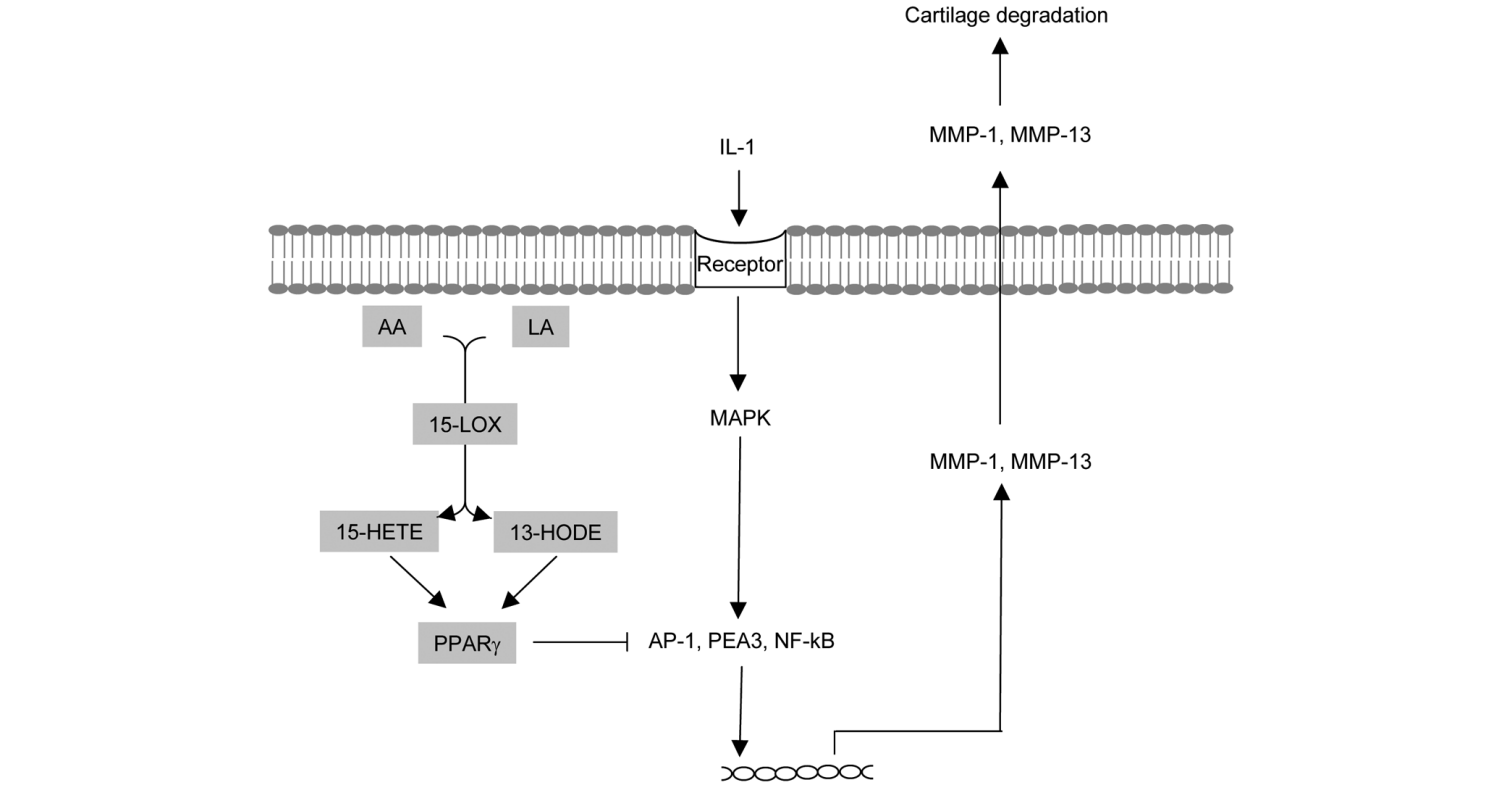
Schematic representation of the suppressive effect of 15-LOX metabolites on MMP-1/MMP-13 expression. Pro-inflammatory cytokines such as IL-1 interact with their respective receptors that activate MAPK signalling and downstream transcription factors, resulting in the transcription of MMP-1 and MMP-13 genes. 15-LOX convert AA and LA to 15-HETE and 13-HODE, which then activate PPARγ. Activated PPARγ antagonizes the transcriptional activity of AP-1, NF-κB and PEA3, which results in the inhibition of the expression of their target genes (for instance, MMP-1 and MMP-13). AA, arachidonic acid; AP, activator protein; HETE, hydroxyeicosatetraenoic acid; HODE, hydroxy octadecadienoic acid; LA, linoeic acid; LOX, lipoxygenase; MAPK, mitogen-activated protein kinase; MMP, matrix metalloproteinase; NF-κB, nuclear factor-κB; PEA3, Polyoma Enhancer Activator 3; PPAR, peroxisome proliferator-activated receptor.

13-HODE and 15-HETE are potent endogenous activators and ligands for PPARγ [23,24]. Using a PPRE reporter plasmid in transient transfection experiments, we confirmed the capability of the above 15-LOX products to activate PPARγ in human chondrocytes. We also showed that pretreatment with an irreversible pharmacological PPARγ antagonist GW9662 overcame the inhibitory effect of 13-HODE and 15-HETE on IL-1β-induced MMP release. These results are consistent with previous findings showing that PPARγ activation suppresses MMP production in several cell types, including chondrocytes [26] and synovial fibroblasts [27]. Altogether, these data strongly suggest that 13-HODE and 15-HETE suppress IL-1β-induced MMP-1 and MMP-13 by chondrocytes through activation of PPARγ. The expression of MMP-1 and MMP-13 are essentially regulated by the transcription factors activator protein (AP)-1 and nuclear factor-κB (NF-κB), and analysis of the 5'-flanking regions of these genes has demonstrated the presence of numerous putative binding sites for AP-1 and NF-κB [3]. On the other hand, previous studies showed that activation of PPARγ suppresses the transcriptional activity of AP-1 and NF-κB [48]. Therefore, it is possible that activation of PPARγ by 13-HODE and 15-HETE reduces transcriptional activity of AP-1 and NF-κB, leading to diminished production of MMP-1 and MMP-13 (Figure 7). Another possible mechanism through which 13-HODE and 15-HETE may downregulate MMP expression could involve the promotion of mRNA decay. Indeed, 15-LOX metabolites were reported to downmodulate lipopolysaccharide-induced TNF-α expression by enhancing mRNA decay [19]. Alternatively, 15-LOX products could prevent IL-1β-induced MMP-1 and MMP-13 expression by interfering with key signalling pathways. In this context, 15-LOX metabolites were shown to inhibit protein kinase C activity and translocation [20,49], and protein kinase C was shown to contribute to MMP-1 and MMP-13 expression [50,51].

15-HETE and 13-HODE are synthesized by a number of cell types such as macrophages, neutrophils and chondrocytes [52]. They have also been detected *in vivo *in several pathophysiological fluids, including sputum from chronic bronchitis patients [53], cerebrospinal fluid from patients with Alzheimer's disease [54], bronchoalveolar lavage fluids from patients with asthma [55] and scleroderma lung disease [56]. Apart from a report by Walenga and coworkers [57], who found that the levels of 15-HETE increase to about 1 μmol/l in blood stimulated with various agents, the concentrations of 15-HETE and 13-HODE detected in most pathophysiological fluids (1 to 100 nmol/l) were lower than those used in the present study (1 to 50 μmol/l). However, it should be noted that, like other eicosanoids, 13-HODE and 15-HETE function as autocrine and paracrine molecules and can readily reach pharmacological levels in the microenvironment of cells that produce them. Moreover, synovial fibroblasts [58] and osteoblasts [59] express 15-LOX and may represent additional sources for the production of 15-LOX metabolites within the joint. Also, we cannot exclude the possibility that low concentrations of 13-HODE and 15-HETE can synergize with each other or with other 15-LOX derivatives to suppress inflammatory and catabolic responses in the joint.

## Conclusions

We demonstrated that 15-LOX-1 and -2 are expressed in OA articular chondrocytes. Treatment with 13-HODE and 15-HETE, the respective metabolites of 15-LOX-1 and -2, suppressed IL-1β-induced MMP-1 and MMP-13 production. These effects do not require protein synthesis and are mediate by PPARγ. These data suggest that 15-LOXs and their metabolites may have therapeutic promise in OA by preventing the production of cartilage-degrading enzymes.

## Abbreviations

AP: activator protein; C_T_: threshold cycle; DMEM: Dulbecco's modified Eagle's medium; ELISA: enzyme-linked immunosorbent assay; FCS: fetal calf serum; GAPDH: glyceraldehyde-3-phosphate dehydrogenase; HETE: hydroxyeicosatetraenoic acid; HODE: hydroxy octadecadienoic acid; IL: interleukin; LOX: lipoxygenase; MMP: matrix metalloproteinase; NF-κB: nuclear factor-κB; OA: osteoarthritis; PBS: phosphate-buffered saline; PCR: polymerase chain reaction; PPAR: peroxisome proliferator-activated receptor; PPRE: peroxisome proliferator-activated receptor-responsive element; SD: standard deviation; TNF: tumor necrosis factor; UNG: uracil-N-glycosylase.

## Competing interests

The authors declare that they have no competing interests.

## Authors' contributions

NC conceived the study, designed and carried out cell and real-time reverse transcription PCR experiments and some immunohistochemistry experiments. NZ contributed to the study design, carried out immunoassays and some cell experiments. MB participated in the study design and data analysis. JM-P, J-PP and ND helped to obtain tissues, and participated in the study design and in some immunohistochemistry experiments. HF conceived, designed and coordinated the study, carried out some cell experiments, and drafted the manuscript. All authors read and approved the final manuscript.
